# Air Pollutants Reduce the Physical Activity of Professional Soccer Players

**DOI:** 10.3390/ijerph182412928

**Published:** 2021-12-08

**Authors:** Michał Zacharko, Robert Cichowicz, Marcin Andrzejewski, Paweł Chmura, Edward Kowalczuk, Jan Chmura, Marek Konefał

**Affiliations:** 1 Department of Biological and Motor Sport Bases, University School of Physical Education, 51-612 Wroclaw, Poland; pawel.chmura@awf.wroc.pl (P.C.); jan.chmura@awf.wroc.pl (J.C.); marek.konefal@awf.wroc.pl (M.K.); 2 Faculty of Architecture, Civil and Environmental Engineering, Lodz University of Technology, 90-924 Lodz, Poland; robert.cichowicz@p.lodz.pl; 3 Department of Recreation, Poznan University of Physical Education, 61-871 Poznan, Poland; andrzejewski@awf.poznan.pl; 4 Football Club Hannover 96, 30169 Hannover, Germany; edward.kowalczuk@hannover96.de

**Keywords:** football, Bundesliga, distances covered, high intensity, O_3_, PM, NO_2_, air quality

## Abstract

The aim of the study was to determine the impact of air quality—analyzed on the basis of the model of integrating three types of air pollutants (ozone, O_3_; particulate matter, PM; nitrogen dioxide, NO_2_)—on the physical activity of soccer players. Study material consisted of 8927 individual match observations of 461 players competing in the German Bundesliga during the 2017/2018 and 2018/2019 domestic seasons. The measured indices included players’ physical activities: total distance (TD) and high-intensity effort (HIE). Statistical analysis showed that with increasing levels of air pollution, both TD (F = 13.900(3); *p* = 0.001) and HIE (F = 8.060(3); *p* = 0.001) decrease significantly. The worsening of just one parameter of air pollution results in a significant reduction in performance. This is important information as air pollution is currently a considerable problem for many countries. Improving air quality during training sessions and sports competitions will result in better well-being and sporting performance of athletes and will also help protect athletes from negative health effects caused by air pollution.

## 1. Introduction

As Reche et al. [1] and Fitch [2] indicate inhalation of high concentrations of air pollutants can cause more harm to athletes who undertake intensive training than to the general public. Athletes are especially vulnerable, as their air intake is higher, and many train and compete outdoors for large portions of the day [1]. During intense exercise, athletes can breathe more than 6000 L/h compared with perhaps 4–500 L/h at rest and 1000 L/h with light exercise [2]. It is caused, among other things, by increased lung ventilation during exercise [3]. Inhaling more air through the mouth during exercise causes a bypassing of the nasal filtration mechanisms. Increased air flow velocity transports pollutants deep into the respiratory tract and thus increases the uptake of gaseous pollutants [4], and thus, the concentration levels of individual pollutants in the human body also increase. Despite the studies cited above, there is a scarcity of research on the physical activity of professional athletes in a potentially polluted environment [5] and in particular on soccer players [6].

It is worth noting that the number and frequency of professional football matches played is high. Modern competitive schedules are very demanding, with teams having to play up to 60 matches per season [7]. Each match requires players to be very physically active, which is defined, among other things, by total distance covered (TD) and high-intensity effort (HIE). The average TD in a Bundesliga match ranges from 10.03 ± 0.61 to 11.55 ± 0.70 km, and the number of HIE from 30.33 ± 7.64 to 47.48 ± 12.30 depending on the position on the field [8]. The above parameters are very important because they significantly correlate with match outcome. For example, Andrzejewski et al. [9] found that winning a match correlates with the players of the winning team covering a greater total distance. Elsewhere, both Modric et al. [10] and Chmura et al. [11] are of the view that high-intensity efforts (fast running and sprinting) are some of the most important measures of physical efficiency in football.

As a result of extensive physical activities that soccer players undertake during a match, there is a high probability of physiological changes, one of which may be the appearance of oxidative stress in the respiratory tract. This is the major pathophysiological factor in adverse vascular health effects of air pollution. The presence of oxidative stress in the lungs has also been documented and almost certainly occurs with immediate exposure [12]. Furthermore, oxidative stress increases lipid peroxidation, generation of secondary mediators that enhance oxidative stress-induced damage, and a reduction in levels of the primary lung antioxidant, glutathione [13]. Depletion of low molecular weight antioxidants, such as glutathione, ascorbate, and tocopherol, along with subsequent reductions of cofactors, such as NADPH, may increase the risk and impact of oxidative stress [14]. It should also be highlighted that in previous research markers of airway inflammation [15] and oxidative stress [16,17] have been detected after exposure to PM and to O_3_. Indeed, PM exposure can directly lead to the expression of particles that cause airway wall fibrosis and play a major role in airway obstruction.

The problem of air quality has been noted by the World Health Organization (WHO), which estimated that, in 2012, about 7 million deaths were associated with living in the areas with polluted air. This is a global problem that affects many countries around the world [18,19,20]. Elevated and/or exceeded levels of air pollutants in certain periods and locations also apply to European countries considered highly developed in terms of their economy and industry. In Germany most urban areas still do not meet WHO air quality standards [21]. Although there are various air quality assessment scales, the domestic ones are usually less strict than European standards. Even so, air quality standards or guidelines are often not met. Among the most frequently analyzed and controlled pollutants are: ozone (O_3_), particulate matter (PM), and nitrogen dioxide (NO_2_). The first of these, ozone, is a gas produced by the action of sunlight on hydrocarbons and nitrogen oxides [22] and is detrimental to athletic performance when exposure is high enough. Subsequent respiratory discomfort associated with increased exposure to ozone may cause a reduction in maximum work efficiency and significantly contribute to an increase in the overall perceived exertion [23]. Particulate matter is produced mainly from the combustion of fuels in gasoline and diesel engines, the combustion of wood and fossil fuels, and during construction works [24]. There are different criteria for the division of particulates; however, the most frequently analyzed are particulates such as PM_10_ (with particle diameter below 10 μm), PM_2.5_ (below 2.5 μm), and PM_1_ (below 1 μm) [2]. The smaller the particle, the greater its potential to cause harm because it can penetrate more deeply into the lungs. However, even PM_10_ has a detrimental effect on health, as its combination with sulfur dioxide (SO_2_) and water vapor creates sulfuric acid-coated particles that can settle in the lungs and cause irritation and asthma-like symptoms [22]. Nitrogen dioxide, on the other hand, is a by-product of the combustion of fossil fuels [22]. NO_2_ tends to coexist with PM and usually O_3_, and they are often inhaled simultaneously [2]. As a result, they have a comprehensive effect on the human body, and for this reason, it seems important to analyze all three of the above types of pollutants simultaneously, especially given the fact that the literature lacks studies that show the integrated effect of the three most often described parameters of air pollution on the body of professional athletes.

Numerous studies show a relationship between air pollution and the cardiovascular and respiratory systems [13,25], and adverse changes in biomarkers of physiological and biochemical functions have also been identified [26]. This is directly related to the performance of the athlete’s body and thus to their activity during sports competitions. In order to address this problem and consider the above-mentioned information, it was decided that the aim of the study would be to determine the impact of air quality analyzed on the basis of the model of integrating three types of air pollutants (O_3_, NO_2_, PM_10_) on the physical activity of football players in the Bundesliga. We hypothesize that as the levels of air pollution increase, players’ physical activity worsens.

## 2. Materials and Methods

### 2.1. Match Sample and Data Collection

Match performance data were collected from 461 soccer players competing in the German Bundesliga during the 2017/2018 and 2018/2019 domestic seasons. In every season, the league’s 18 teams face each opponent twice per season, home and away. Thus, a season comprises of 34 match days and 306 matches, typically held on weekends between August and May [6]. A total of 8927 individual match observations were made of outfield players (goalkeepers were excluded). Only data for players completing entire matches (i.e., on the pitch for the whole 90 min) were considered.

Data were obtained using the IMPIRE AG system with a recording frequency of 25 Hz [27]. Each player’s movements were recorded by two cameras [28]. The system utilizes state-of-the-art algorithms and 2-D and 3-D video-recording technology, allowing for detailed motion analysis of entire soccer matches. The major advantages of vision-based systems are their high update rate corresponding to the camera-frame rate and the fact that the players and the ball are tracked simultaneously. The validity and reliability of this system have been described in detail elsewhere [28]. Furthermore, Liu et al. [29] showed that team match events coded by independent operators using this system achieved very good levels of agreement (weighted kappa values of 0.92 and 0.94), with the average difference of event time equal to 0.06 ± 0.04 s.

This study maintains the anonymity of the players following data protection laws, is conducted in compliance with the Declaration of Helsinki, and was approved by the Senate Committee on Ethics of Scientific Research at the Academy of Physical Education in Wroclaw (No. 12/2021).

### 2.2. Procedures

Air quality data was determined on the basis of records from the air pollution monitoring system of the German Environment Agency [30]. This is Germany’s main environmental protection agency and is also a German point of contact for numerous international organizations, such as the WHO. The following air pollution parameters were analyzed: particulate matter smaller than ten micrometers (PM_10_), nitrogen dioxide (NO_2_), and ozone (O_3_) [31]. The data were read from air pollution meters closest to the stadiums where the matches were played (average distance 3.5 km). For each analyzed match, the mean value of air pollution readings was determined from the data recorded at the beginning and end of the match (average over two hours). In the following stage, the average value of the readings for each parameter was classified as very good, good, moderate, sufficient, bad, or very bad, in accordance with the standards adopted in Poland by the Main Inspectorate Of Environmental Protection [32]—Table 1. The above scale makes it possible to accurately assess all tested parameters and is also widely used in scientific research [24,33].

For each “air quality index”, point values were assigned from 1 (very good) to 6 (very bad)—Table 1. Then, based on the sum of point values assigned to individual “air quality index” categories, a model of integrating three types of air pollutants was created. Based on this model, 4 air quality categories were created: “very good”—the sum of the point values for the three tested parameters (PM_10_, O_3_, NO_2_) is 3; “fair”—the sum of the point values for the three tested parameters is 4; “moderate”—the sum of the score values for the three parameters tested is 5; and “poor”—the sum of the point values for the three tested parameters is at least 6.

In the model of integrating selected air pollution parameters, “very good” (2563 observations) means that all three parameters subject to observation had a “very good air quality index” (e.g., 1 + 1 + 1 = 3). For “fair” (3749 observations), one of the parameters had a higher point value, defined in the “air quality index” as “good” (e.g., 2 + 1 + 1 = 4). For “moderate” (1882 observations), for example, one of the parameters had a point value of 1, and the other two parameters had a point value of 2 (e.g., 1 + 2 + 2 = 5). For “poor” (733 observations), many configurations are possible (e.g., 3 + 2 + 1 = 6).

The measured indices included players’ physical activities: total distance (TD, distance covered by a player during match play) and high-intensity effort (HIE, running efforts (velocity > 4 m/s) achieved by a player during match play). Complete definitions of physical variables are available at the Deutsche Fußball Liga (DFL) [34]. Definitionskatalog Offizielle Spieldaten—Bundesliga website https://s.bundesliga.com/assets/doc/10000/2189_original.pdf (accessed on 6 June 2019).

### 2.3. Statistical Analyses

All variables were checked to verify their conformity with a normal distribution. Arithmetic means and standard error were calculated. Spearman’s correlations were used. Then repeated-measures ANOVA was used to compare mean values for the examined variables. Fisher LSD (Least Significant Difference) post-hoc tests were performed to assess differences between means. Moreover, partial eta squared (η^2^) was calculated [35]. All statistical analyses were performed using the Statistica ver. 13.1 software package (Dell Inc., Tulsa, OK, USA).

## 3. Results

The following mean levels and standard errors of air pollution parameters were recorded during the study: PM_10_, 19.04 ± 0.12 (confidence interval −95%—18.80; confidence interval 95%—19.28) (µg/m^3^); O_3_, −56.50 ± 0.32 (confidence interval −95%—55.87; confidence interval 95%—57.13) (µg/m^3^); NO_2_, −36.07 ± 0.37 (confidence interval −95%—35.34; confidence interval 95%—36.79) (µg/m^3^). Value of air pollution parameters for individual air quality categories are shown in Table 2.

We did not find any correlations between the individual air pollution parameters (PM_10_, O_3_, NO_2_) and players’ physical activities (TD, HIE): PM_10_ and TD r_s_ = −0.045; PM_10_ and HIE r_s_ = −0.013; O_3_ and TD r_s_ = −0.108; O_3_ and HIE r_s_ = −0.093; NO_2_ and TD r_s_ = 0.005; and NO_2_ and HIE r_s_ = 0.040. Then, a model was developed that integrated the three types of air pollutants was created.

**Figure 1:**
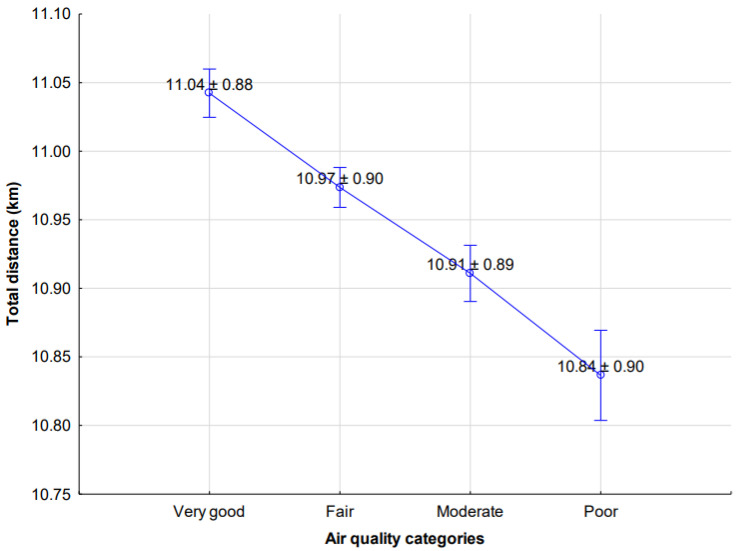
Differences in total distance covered by soccer players in relation to changes in air quality categories (mean ± SE). Differences statistically significant between very good vs. fair *p* = 0.01, fair vs. moderate *p* = 0.01, and moderate vs. poor *p* = 0.05 (Source: own elaboration).

**Figure 2:**
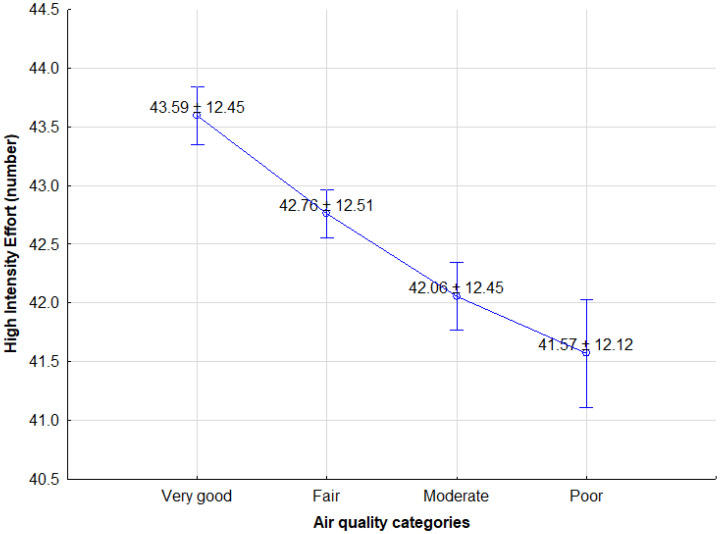
Differences in high-intensity effort of soccer players in relation to changes in air quality categories (mean ± SE). Differences statistically significant between very good vs. fair *p* = 0.01 and fair vs. moderate *p* = 0.05 (Source: own elaboration).

The statistical analysis of player’s physical activities as set against air quality categories (very good, fair, moderate, poor) revealed effects in relation to the TD (F = 13.900(3); *p* = 0.001; η^2^ = 0.005), as shown in Figure 1, and HIE (F = 8.060(3); *p* = 0.001; η^2^ = 0.003), as shown in Figure 2.

## 4. Discussion

The study aimed to determine the impact of air quality—analyzed on the basis of the model of integrating three types of air pollutants (O_3_, NO_2_, PM_10_)—on the physical activity of soccer players in the Bundesliga. So far, little research has been undertaken in this field in relation to athletes. Rundell [5] analyzed ice hockey players and skaters and identified that PM products from diesel-powered Zambonis (ice resurfacing machines used in ice rinks) was a factor in the increased prevalence of asthma and airway hyperresponsiveness. His research identified that even warming-up while breathing polluted air containing PM could reduce exercise performance. Whilst that study concerned indoor sport, studies on outdoor sports, where the analysed material is derived from mostly runners or cyclists, are more popular. For example, El Helou et al. [36] found that higher ozone levels were associated with poorer performance in six city marathons. Reduced lung function has also been observed among runners after they ran near busy highways [17] and among cyclists after they rode along heavy traffic routes during rush-hour traffic [37].

Our study is both original and important because, thus far, to the best of our knowledge, only one study on the effects of air quality on the activity of professional soccer players has been published. This is surprising because soccer is one of the most popular outdoor sports; according to the latest FIFA Professional Football Report, it is played by over 128,000 professional football players from 187 countries [38]. In the study concerned, Lichter et al. [6] assessed the effects of particulate air pollution on soccer players in German stadiums, revealing that performance was reduced under poor air quality conditions. In that study, PM_10_ was the only factor taken into account, and the productivity indicator was limited to only the number of passes during a match. However, total distance covered and high intensity effort seem to be more valuable parameters for assessing performance in the context of air quality because soccer is considered a high-intensity, intermittent sport with an unprecedented increase (up to 50%) in high-impulsive actions occurring during match play [39]. Moreover, high-intensity running and accelerations are nowadays the most crucial activities where elite soccer-match performance is concerned [40]. Therefore, in our analysis, the range of measured parameters of air pollutants was significantly expanded, and it was decided that physical activities be included too. Thanks to this, we were able to discover that lowering the air quality level during the match not only negatively affects technical activities, such as passes [6], but also the most important physical activities, such as TD and HIE. Our analysis shows that each level of air quality deterioration significantly reduces both the TD covered by the players and the number of HIE. The difference between the average distance travelled by each player in matches with “very good air quality” and “poor air quality” is 0.2 km (approximately 2 km for the entire team), and the difference in the number of HIEs performed is two repetitions, i.e., as much as 20 for the entire team, which may indirectly impact the game result [11].

In our study, in conditions of “poor air quality” footballers covered the distance 10.84 ± 0.90 km and performed 41.57 ± 12.12 number of HIE. Duda et al. [41] claimed that during enhanced physical activity, athletes inhale up to 20 times more air than during a walk, and a great amount of toxic substances enter their bodies and poison the body’s tissue. The greater amount of particulate matter and nitrogen dioxide that enter the body with polluted air may increase bronchiolar fibrosis and play a major role in airway obstruction [42,43]. Exposure to ozone, on the other hand, impairs the function of the endothelium of the ductal vessels [44]. This can result in, amongst other things, endogenous airway acidification episodes indicative of pollution-related lung inflammation [45]. Furthermore, a study by Kargarfard et al. [25] showed that a high concentration of pollutants during physical activities slows cardiovascular functions as well as impacting hematological parameters. Thus, air quality during a football match is extremely important because, together with increased physical effort, the body absorbs harmful substances from the air [46]. Each of the selected air pollutants included in our study (PM_10_, O_3_, NO_2_) causes other negative effects in the human body. However, all three of them are particularly dangerous, which is confirmed by the research by Rundell et al. [13] and Tainio et al. [31].

In view of the harmful effects of air pollution on the human body, it is worth considering what should be done to improve air quality at stadiums and how not to expose athletes and fans to negative health effects. One possible way in which better natural environments might aid health and athletic performance is through mitigation of risk from environmental pollutants. Existing research on this topic has shown that trees can reduce the level of air pollutants in urban areas [47], with one study claiming that in the U.S., trees remove 711,000 tons of air pollutants per year [48]. With regard to athletes, De Wolfe et al. [49] looked into the performance of 128 college-level track and field competitors across four locations that differed in their greenness. The authors found that greenness was a predictor of performance (r^2^ = 0.61, *p* < 0.001), with athletes’ best performances being more likely to occur at the most “green” site. Therefore, it is worth paying attention to having as much greenery as possible in the vicinity of football stadiums. At sites where substantial tree planting in surrounding areas is not possible, roof spaces can be used to green the area. Yang et al. [50] estimated that 1675 kg of air pollutants were removed by 19.8 hectares of green roofs in one year in Chicago. Another way is to install various types of air purification filters near stadiums, which are able to reduce the concentration of particulates in the air by up to about 2 million milligrams per month [51]. By applying the above solution, air pollution during matches will likely be reduced, and both footballers and fans will be less exposed to the negative health effects caused by poor air quality.

The authors are fully aware of the many factors that might have influenced the results of the analyses presented here [52]. The monitoring location was determined at the measuring stations closest to the stadiums. However, to make the measurements more precise, in subsequent analyses, we suggest placing air pollution meters directly next to the stadiums. In addition, the meteorological conditions and the type of buildings between the measuring station and the stadium were not taken into account, and this may have an impact on the actual air quality in a particular stadium. A further limitation concerns the failure to take account of many other parameters helping to characterize external load, such as player load, sprints, acceleration, deceleration, impact of age, and positions, which all also serve to express the demands imposed by matches in non-cyclic team sports. It should also be noted that the linear associations with the individual pollutants (PM_10_, O_3_, NO_2_) were not significant. This may be due to the above-mentioned limitations, and therefore, more research is needed to properly assess the individual pollutant values.

## 5. Conclusions

An important new finding of the present study is that air quality significantly impacts the physical activity of soccer players. As air pollution levels increase, physical activity during a match decreases. Worsening of just one parameter of air pollution is enough to result in a significant reduction in performance, and this may be a consequence of negative physiological reactions in the body. This is an important finding because air pollutants are currently a significant problem in many countries, and our study is further evidence that action should be taken to improve air quality. Improving air quality during training and sports competitions will result in better well-being and sporting performance of athletes and will also help protect athletes from the negative health effects caused by air pollution.

## Figures and Tables

**Table 1:** Inspectorate of Environmental Protection (Poland) norms for hourly concentrations and assigned point values.

Air Quality Index	Air Pollution Parameter			
	PM_10_ (µg/m^3^)	O_3_ (µg/m^3^)	NO_2_ (µg/m^3^)	Points
Very good	0–20	0–70	0–40	1
Good	20.1–50	70.1–120	40.1–100	2
Moderate	50.1–80	120.1–150	100.1–150	3
Sufficient	80.1–110	150.1–180	150.1–200	4
Bad	110.1–150	180.1–240	200.1–400	5
Very bad	>150	>240	>400	6

**Table 2:** Value of air pollution parameters for individual air quality categories (mean ± SE).

Air Quality Index	Air Pollution Parameter			
	PM_10_ (µg/m^3^)	O_3_ (µg/m^3^)	NO_2_ (µg/m^3^)	Points
Very good	12.51 ± 0.06	40.47 ± 0.25	19.56 ± 0.14	1
Good	29.28 ± 0.14	86.73 ± 0.26	57.80 ± 0.25	2
Moderate	59.92 ± 0.43	132.47 ± 0.57	116.72 ± 1.13	3
Sufficient	-	-	-	4
Bad	-	-	272.58 ± 4.33	5
Very bad	-	-	-	6

## Data Availability

The data used in this research were acquired from a third party: https://matchanalysishub.bundesliga.com/login (accessed on 6 June 2019). The data were provided as part of a scientific cooperation agreement with a professional football club that plays in the 2. Bundesliga. In line with ethical approval for the research, the authors are also prevented from sharing any data that could be re-identified, as a combination of the metadata and the score data would allow for teams, and possibly also players, to be re-identified. However, access to these data is possible from the third-party. The data acquired for this investigation were so-called “excel dumps” of player statistics for each match during the 2017–18 and 2018–19 seasons. Access to the data can be sought via Match Analysis Hub: mdc@sportec-solutions.de.
